# Nonspecific periaqueductal gray lesions on T2WI in episodic migraine

**DOI:** 10.1186/s10194-016-0695-9

**Published:** 2016-10-28

**Authors:** Zhiye Chen, Xiaoyan Chen, Mengqi Liu, Shuangfeng Liu, Lin Ma, Shengyuan Yu

**Affiliations:** 1 Department of Radiology, Chinese PLA General Hospital, 28 Fuxing Road, Beijing, 100853 China; 2 Department of Neurology, Chinese PLA General Hospital, 28 Fuxing Road, Beijing, 100853 China

**Keywords:** Episodic migraine, Periaqueductal gray, Hyperintensity lesions, Magnetic resonance imaging

## Abstract

**Background:**

The specific periaqueductal gray (PAG) lesions with migraine-like headache were easily identified on conventional MR images in clinical practice, and the aim of this study is to investigate the nonspecific periaqueductal gray (PAG) lesions in episodic migraine (EM) patients based on T2 weighted imaging (T2WI).

**Methods:**

T2WI images were obtained from 18 EM patients and 18 normal controls (NC) on 3.0 T MR system. The images were observed by two experienced radiologists, and the lesions were identified on T2WI by consensus by 2 experienced neuroradiologists blinded to the patient identity. Chi-Square test was performed for the significance test.

**Results:**

Ring-like hyperintensity lesions (HILs) around the PAG region were observed in 14 EM patients and in 5 NCs on T2WI. Four EM patients and 13 NCs were normal in PAG region. The significance was revealed by Chi-Square test (*P* = 0.003).

**Conclusion:**

HIL of PAG may be the direct evidence of the EM genesis, and the further structural and functional study should be performed to elucidate the neuromechanism of migraine pathogenesis.

## Background

Periaqueductal gray (PAG) was a center with powerful descending antinociceptive neuronal network, which include various layered neurons around the aquaeductus mesencephali [1, 2]. PAG dysfunction was recognized in migraine [3], and the PAG dysfunction may be associated with iron deposition and may be considered as a possible “generator” of migraine attacks [1, 4, 5].

The PAG lesions with migraine-like headache were mainly identified in multiple sclerosis [6–10] and infarction [11]. These lesions could be considered as specific lesions because of definite clinical diagnosis. Brain T2-visible hyperintensity lesions commonly presented in migraine patients [12, 13], and these T2 lesions may be seen in gray matter, and commonly located in white matter. However, T2-visible PAG lesions were not reported up to now. Compared with the previous reported specific lesions, the T2-visible lesions could be called as nonspecific lesions.

Above referred to specific PAG lesions could lead to migraine or migraine-like symptoms, herein, we hypothesize that non-specific PAG lesions on T2WI may present in the migraine and may be the direct evidence of migraine. To address this hypothesis, we prospectively obtained oblique axial T2WI data from 18 EM patients, and observed the signal and appearance of the T2-visible lesions on PAG region in EM patients, and the same observation was performed in NCs.

## Methods

### Subjects

Written informed consent was obtained from all participants according to the approval of the ethics committee of the local institutional review board. Eighteen EM patients were recruited from the International Headache Center, Department of Neurology, Chinese PLA General Hospital. All the following inclusion criteria should be fulfilled: 1) EM is defined as migraine attack days being less than 15 days per month. The definition of migraine refers to 1.1 Migraine without aura and 1.2 Migraine with aura in ICHD 3beta [14]; 2) no migraine preventive medication used in the past 3 months; 3) age between 20 and 60 years; 4) right-handed; 5) absence of any chronic disorders, including hypertension, hypercholesterolemia, diabetes mellitus, cardiovascular diseases, cerebrovascular disorders, neoplastic diseases, infectious diseases, connective tissue diseases, other subtypes of headache, chronic pain other than headache, severe anxiety or depression preceding the onset of headache, psychiatric diseases, etc.; 6) absence of alcohol, nicotine, or other substance abuse; and 7) patient’s willingness to engage in the study. Eighteen NCs were recruited from the hospital’s staff and their relatives. Inclusion criteria were similar to those of patients, except for the first two items. NCs should never have had any primary headache disorders or other types of headache in the past year. General demographic and headache information were registered and evaluated in our headache database. Additionally, we evaluated anxiety, depression, and cognitive function of all the participants by using the Hamilton Anxiety Scale (HAMA) [15], the Hamilton Depression Scale (HAMD) [16], and the Montreal Cognitive Assessment (MoCA) Beijing Version (www.mocatest.org). The exclusion criteria were the following: cranium trauma, illness interfering with central nervous system function, psychotic disorder, and regular use of a psychoactive or hormone medication. The study protocol was approved by the Ethical Committee of Chinese PLA General Hospital and complied with the Declaration of Helsinki. Informed consent was obtained from all participants before the study. MRI scans were taken in the interictal stage at least three days after a migraine attack for EM patients. All the patients were given with the Visual Analogue Scale (VAS) and the Migraine Disability Assessment Scale (MIDAS). All the subjects were right-handed and underwent conventional MRI examination to exclude the subjects with cerebral infarction, malacia, or occupying lesions. Alcohol, nicotine, caffeine, and other substances were avoided for at least 12 h before MRI examination.

### MRI acquisition

Images were acquired on a GE 3.0 T MR system (DISCOVERY MR750, GE Healthcare, Milwaukee, WI, USA) and a conventional eight-channel quadrature head coil was used. All subjects were instructed to lie in a supine position, and formed padding was used to limit head movement. Oblique axial T2-weighted imaging (T2WI)(TR = 5000 ms, TE = 113.4 ms, FOV = 24 cm × 24 cm, Matrix = 384 × 384, slice thickness = 6.0 mm, slice space = 1.0 mm) were obtained. Besides these, T1 fluid-attenuated inversion recovery (T1-FLAIR) and diffusion weighted imaging (DWI) were also acquired. The T1-FLAIR parameters included: TR/TE = 2040 ms/6.9 ms, FOV = 24 cm × 24 cm, Matrix = 384 × 256, slice thickness = 6.0 mm, slice space = 1.0 mm. DWI parameters included: TR/TE = 6000 ms/65.7 ms, FOV = 24 cm × 24 cm, matrix = 192 × 192, slice thickness = 6.0 mm, slice space = 1.0 mm) with b = 1000 s/mm2 applied in the x, y, and z directions and b = 0 s/mm2 without motion-probing gradients, followed by automatic generation of isotropic DWI. All the images had the same location, FOV, slice thickness and slice space.

## MR image review

All the T2WI data were reviewed on the picture archiving and communication system (PACS) workstation. MR images were reviewed with the same film layout, magnification, and field of view, without the names of the subjects. The imaging findings were blindly reviewed in consensus by two experienced neuroradiologists without knowing the clinical information.

## Definition of MR signs in PAG region on T2WI

**Fig. 1:**
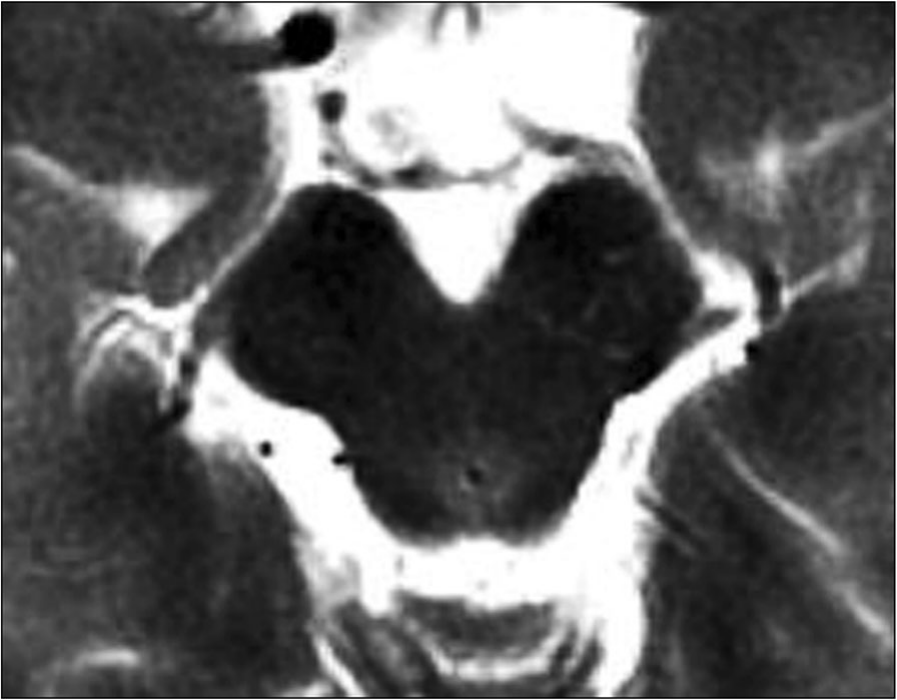
Ring-like slight hyperintensity lesion (ring sign) around the aquaeductus mesencephali, and the flow void in the aquaeductus mesencephali could be seen

**Fig. 2:**
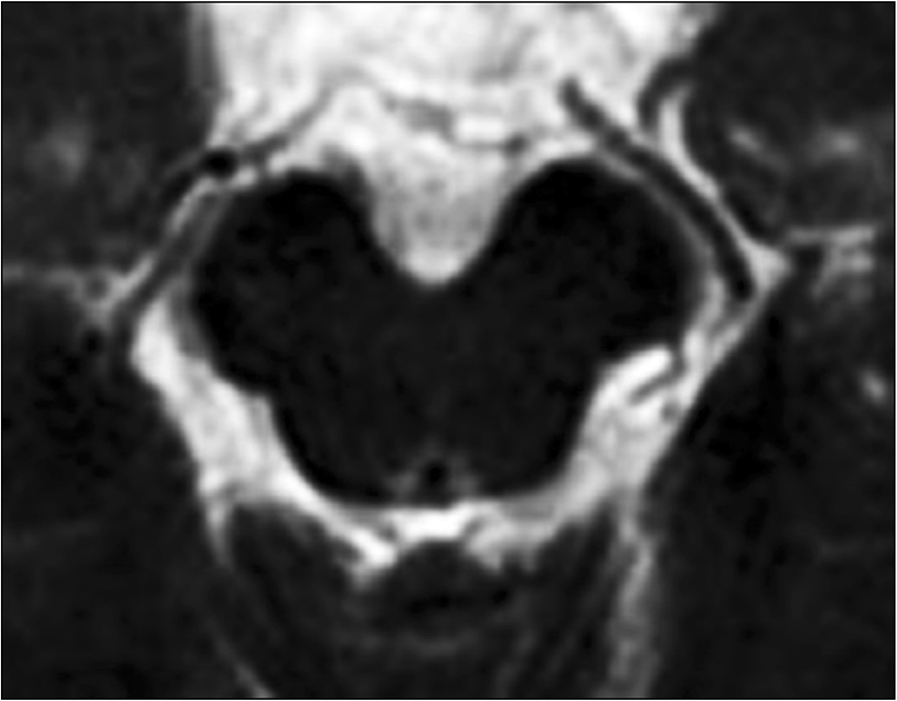
Irregular ring-like hyperintensity (dot-ring sign) around the aquaeductus mesencephali

Hyperintensity lesions (HIL) (Figs. 1 and 2) was defined as follows: (1) the lesion’s signal was higher compared with normal brain tissue; (2) the lesion around the aquaeductus mesencephali; (3) the lesion with ring-like slight hyperintensity could be called as ring sign, and the lesion with irregular ring-like could be called as dot-ring sign with least three dots in the ring. (4) the lesion was not visible on T1WI and DWI; (5) no abnormal signal except PAG region. The exclusion criterion included: (1) acute infarction in the PAG region or peri-PAG region which would be visible on DWI; (2) mass lesion in the PAG region or peri-PAG region; (3) inflammatory demyelinating lesions in the PAG region or peri-PAG region; (4) multiple supratentorial abnormal signal on T2WI.

## Statistical analysis

The statistical analysis was performed by using PASW Statistics 18.0. The significance differences of HIL between EM group and NC group were computed using Pearson Chi-Square test. The Spearman’s correlation analysis was applied between migraine duration and positive PAG lesions on T2WI. Significant difference was set at a *P* value of < 0.05.

## Results

### Demography and neuropsychological test

Demographic and clinical data are summarized in Table 1. Eighteen EM patient (F/M = 14/4) and 18 age and sex-matched NCs (F/M = 14/4) were enrolled. There was no significant difference for age between EM (33.39 ± 10.69 years old) and NC (33.16 ± 10.77 years old). The scores of HAMA, HAMD and MoCA were 15.67 ± 9.85, 10.89 ± 7.26 and 29.17 ± 1.47, respectively. The disease duration was 12.44 ± 8.07 years, and the scores of VAS and MIDAS were 8.33 ± 1.50 and 16 ± 17.94, respectively.

**Table 1:** The clinical characteristics of EM patients and health cases (normal control, NC)

	EM	NC
Num (F/M)	18 (14/4)	18 (14/4)
Age*	33.39 ± 10.69	33.16 ± 10.77
HAMA*	15.67 ± 9.85	NA
HAMD*	10.89 ± 7.26	NA
MoCA*	29.17 ± 1.47	NA
DD (yrs)	12.44 ± 8.07	NA
VAS	8.33 ± 1.50	NA
MIDAS	16 ± 17.94	NA

*NA* Not available, *DD* disease duration

*There was no significant between EM and NC (*P* > 0.05)

### Comparison of HIL between EM group and NC group

HIL was observed in 14 EM patients (14/18) and was observed in 5 NCs (5/18). The Chi-Square test demonstrated there was significant difference between EM and NC group (*P* = 0.003) (Table 2).

**Table 2:** Comparison of HIL of PAG between EM and NC

T2 lesion	EM	NC	Chi-Square Value	*P* value
Positive	14	5	9.028	0.003
Negative	4	13		

### Correlation analysis between migraine duration and PAG lesion

Spearman’s correlation analysis showed that there was no significant correlation between migraine duration and PAG lesion on T2WI (*P* = 0.347).

## Discussion

Migraines are a common type of primary headaches with a reported prevalence of approximately 5.7 % in men and 17.0 % in women [17], and affect 12 % of the population worldwide [18]. In China, the prevalence of migraine is 9.3 % of the general population [19]. Migraines are also a major cause of chronic headaches, with approximately 2.5 % of EM transformed to new-onset chronic migraine [20]. Therefore, the neuromechanism of migraine has been the key focus of research [21].

The current MRI study about the role of PAG in migraine genesis mainly focused on the voxel-based morphometry [12], resting-state functional MRI [22, 23], diffusion kurtosis imaging [24], positron emission tomography [25], and ion deposition study [1]. Conventional MRI studies reported the specific T2 lesions in PAG region which lead to migraine or migraine-like symptoms, and non-specific T2 lesions in PAG region was still not revealed up to now. In this study, we prospectively observed the MR appearance of PAG on the T2WI. The findings were interesting, and the ring-like HILs were identified in most of the cases. Further statistical analysis demonstrated that there was significant difference between EM and NC group. Therefore, it was reasonable to assume that HIL might be the major feature of EM on conventional MR imaging.

A previous study [12] demonstrated that gray matter density in PAG was increased compared with normal controls, and these findings may be associated with the signal increased in T2WI, which reflect the hydrogen proton increasing. HILs of PAG region also indicated that axons in this region might undergo degeneration, which might be associated with migraine attack [12]. Besides, ion deposition load may aggravate the PAG burden, and then lead to the dysfunction of PAG [1].

Spearman’s correlation analysis also demonstrated that there was no significant correlation between migraine duration and PAG lesions, which was not consistent with the previous study [1]. In this study, positive correlation was found for duration of illness with R2' (transverse relaxation rates reflecting ion deposition extent) in the EM group. The R2' change may indicate PAG dysfunction, and it was not visible on T2WI. While HIL was visible on T2WI, and no correlation with migraine duration may suggest that nonspecific PAG lesion would be an intrinsic changes for EM. Besides, it may be associated with the small sample size.

In this study, HILs included ring sign and dot-ring sign, which may be have different pathophysiological mechanism. For the ring sign, it commonly was around the aqueductus mesencephali, and showed slight homogeneous hyperintensity. Based on the previous documents, it may be associated the ion deposition [1], frequent episodic activation [26], increased mean kurtosis and mean diffusivity value based on diffusion kurtosis imaging (DKI) [24] and altered functional connectivity [22], and this sign may be the direct imaging response for the intrinsic functional changes of PAG. While dot-ring sign always encircled the aqueductus mesencephali, and it may be associated with the ependyma [27], which would facilitates movement of iron, bound to ferritin, into brain and the neuron by the transferrin transporter [28–30]. Herein, the exact pathophysiology mechanism should further be investigated.

In the clinical practice, the specific lesions in PAG could easily be noticed while the non-specific lesion such as HILs commonly might be neglected. This study provided the direct imaging evidence for the change of PAG region in EM, and it was also easy to be identified on the T2WI, and could be applied with the routine MRI examination. The main limit of this study was that the sample of this study was relative small, and it would be necessary to increase the sample size in the future study.

## Conclusion

In conclusion, HIL was a non-specific T2 lesion of PAG, and it might be the predominant MR hallmark and evidence for EM patients.

## Abbreviations

EM: Episodic migraine
HIL: Hyperintensity lesion
NC: Normal controls
PAG: Periaqueductal gray
T2WI: T2 weighted imaging
